# Complete chloroplast genome of *sageretia thea* (rhamnaceae), an ornamental fruit and medicinal tree

**DOI:** 10.1080/23802359.2024.2329667

**Published:** 2024-03-26

**Authors:** Misha Zhan, Xiaoyu Wang, Wenyue Chen, Xiaoling Huang

**Affiliations:** a Jiyang College, Zhejiang A&F University, Zhuji, China; b Hangzhou Vocational & Technical College, Hangzhou, China

**Keywords:** *Sageretia thea*, chloroplast genome, phylogenetic analysis

## Abstract

*Sageretia thea* (Osbeck) M.C. Johnst (1968) is an important fruit and medicinal species of Rhamnaceae family. The complete chloroplast genome (cp) of *Sageretia* genus was sequenced and reported for the first time in this study. The cp genome had a total length of 161,352 bp, consisting of a largesingle-copy (LSC) region of 89,802 bp, a small single-copy (SSC) region of 18,914 bp, and a pair of inverted repeat (IR) regions totaling 26,318 bp. The plastid genome contained 129 genes, including 84 protein-coding genes, 37 tRNA genes, 8 rRNA genes. The overall GC content of the genome was found to be 37.10%. Phylogenetic analysis based on comparison with 27 chloroplast genomes revealed that *S. thea* is closely related to genera *Rhamnus* and *Berchemia.* The findings of this study can provide fundamental insights for the conservation, exploitation, and systematic genomic investigation of Rhamnus plants.

## Introduction

*Sageretia* plants, belonging to Rhamnaceae family and first discovered in 1826, have been documented with over 40 species. It is primarily distributed in southern and eastern Asia, with a few species found in the Americas and Africa. China alone accounts for 22 of these species. The majority of *Sageretia* plants possess high economic and horticultural value, *Sageretia thea* (*S. thea*) was one of the most significant fruit and medicinal plant. The underground part of *S. thea* possesses properties such as expectorant, carminative, and dehumidifying effects. Previous studies on *S. thea* have primarily focused on its fruit yield and medicinal ingredients (Sang et al. 2015; Khalil et al. 2021), with limited attention given to its distribution and evolutionary relationships within the *Sageretia* genus. To date, there is no complete chloroplast genome available for *Sageretia* species, which could provide valuable information for exploring phylogenetic relationships within the Rhamnaceae family and serve as a foundation for future research.

## Materials and methods

**Figure 1.**
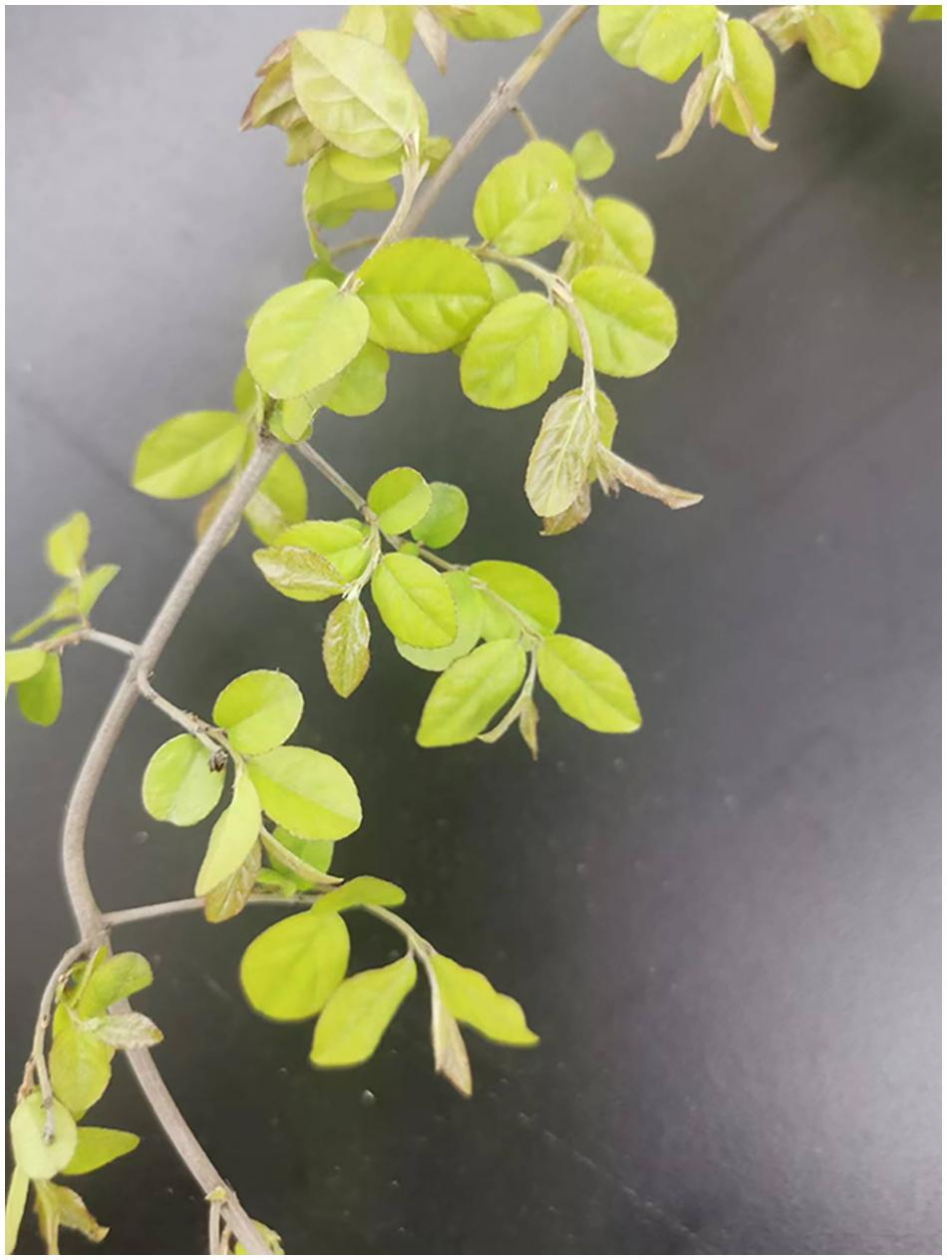
The reference image of the plant of *sageretia thea*(taken by *misha zhan,* photographed in the Mountain slope regions of the Zhejiang province, China; the most characteristic feature of the specimen: Rattan shrub, leaves subopposite or alternate, elliptic, margin serrate).

Fresh leaves of *S. thea* were collected from wild plants grown in Dongbai Mountain, China (116°41’N, 39°91’E, Figure 1). The certificate specimens were deposited in Herbarium, College of Life Sciences, Northwest A&F University (https://www.cvh.ac.cn/spms/detail.php?id=bf709a81, Zhenhai Wu, wzhhai@nwsuaf.edu.cn) under the registration number of 0295848. The fresh leaves were frozen in liquid nitrogen and stored at −80 C until further use. Total genomic DNA was extracted using Doyle’s (1987) method. Paired-end reads of 150 bp were generated using illumina NovaSeq 6000 platform (Illumina, San Diego, CA). SPAdes v3.10.1 software was used to de novo assembly the cp genome (Bankevich et al. 2012; Kongkachana et al. 2022). The fastp v0.20.0 (https://github.com/opengene/fastp) software were used to filter the raw data, the filtration criteria were as follows: (1) truncate the sequencing linker in the Reads and the primer sequence (2) filter out the Reads whose average quality value is less than Q5, (3) filter out N Reads whose number is more than 5. The de novo assembly was performed using filtered reads. The clean data consisted of a total base count of 5,300,864,700 with a percentage mass value greater than or equal to 20 up to 96.75%. The cp genome of *Berchemia flavescens* (GenBank accession MK460212.1) was used as the reference genome for quality control in this study (Figure S1). Twenty six plastid genomes of Rhamnaceae family were chosen to draw the phylogenetic tree, *Vitis davidii* and *Vitis amurensis* as outgroups. The sequences used in this study were downloaded from NCBI GenBank. The cp genome of *S. thea* were aligned with 27 species belonging to Rhamnaceae family from the same starting point using MAFFT v7.427 (auto mode). The evolutionary tree was build using RAxML v8.2.10 (https://cme.h-its.org/exelixis/software.html) software, chosen GTRGAMMA model and set bootstraps as 1,000 based on the rapid Bootstrap analysis.

## Results

**Figure 2.**
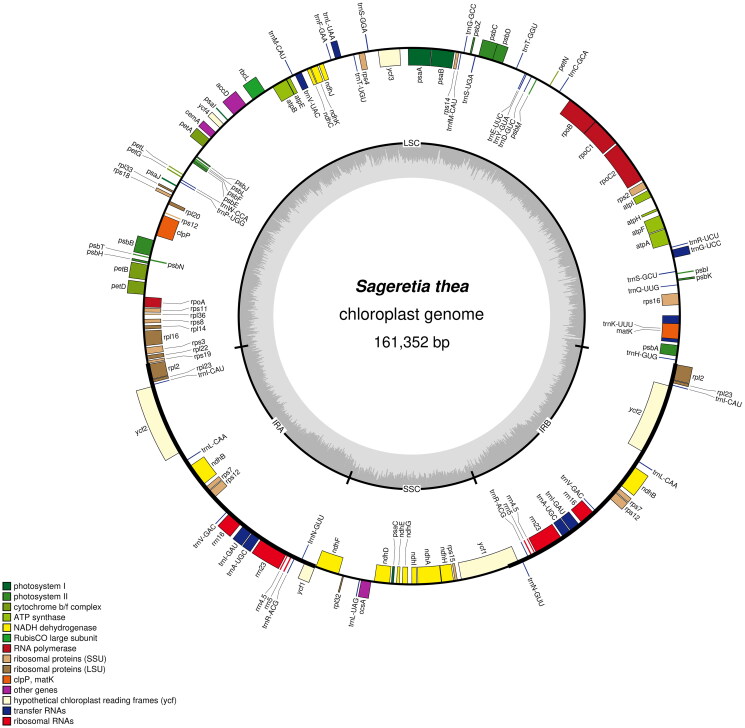
Gene map of the *sageretia thea* cp genome. The four areas (SSC, LSC, IRA and IRB) had been noted in black coil. Genes reside in the inside and outside of the outer circle are in the forward and reverse directions, respectively. The dark and light gray bars in the inner circle denote G + C and a + T contents, respectively.

**Figure 3.**
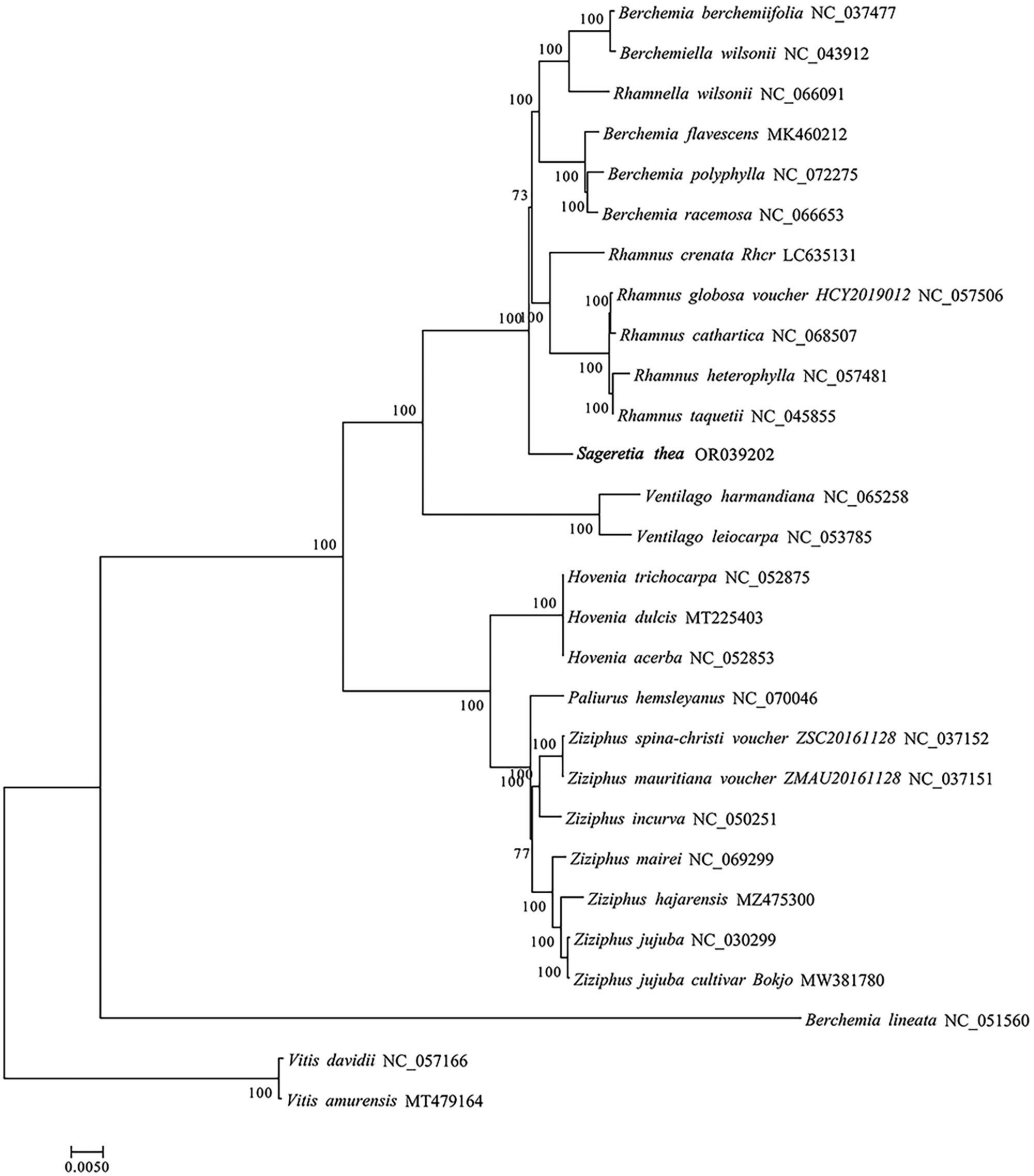
Phylogenetic tree of 26 species of rhamnaceae family based on complete chloroplast genomes. Two taxa (*vitis davidii* and *vitis amurensis*) as outgroups. The species are: *Sageretia thea* (OR039202), *berchemia berchemiifolia* (NC_037477; Cheon et al. 2018), *berchemiella wilsonii* (NC_043912; Li et al. 2019), *rhamnella wilsonii* (NC_066091), *berchemia flavescens* (MK460212; zhu et al. 2019), *berchemia polyphylla* (NC_072275), *berchemia racemosa* (NC_066653), *rhamnus crenata* (LC635131), *rhamnus globosa* (NC_057506; Xie et al. 2020), *rhamnus cathartica* (NC_068507), *rhamnus heterophylla* (NC_057481; Li et al. 2020), *rhamnus taquetii* (NC_045855; jin et al. 2020), *ventilago harmandiana* (NC_065258), *ventilago leiocarpa* (NC_053785), *hovenia trichocarpa* (NC_052875; Li et al. 2020), *hovenia dulcis* (MT225403; Li et al. 2020), *hovenia acerba* (NC_052853; Zhang et al. 2020), *paliurus hemsleyanus* (NC_070046), *ziziphus spina-christi* (NC_037152), *ziziphus mauritiana* (NC_037151), *ziziphus incurva* (NC_050251), *ziziphus mairei* (NC_069299), *ziziphus hajarensis* (MZ475300), *ziziphus jujuba* (NC_030299; Ma et al. 2017), *ziziphus jujuba cultivar bokjo* (MW381780), *berchemia lineata* (NC_051560; Xie et al. 2020), *vitis davidii* (NC_057166; Tian et al. 2019), *vitis amurensis*(MT479164; Guo et al. 2020).

The cp genome of *S. thea* which was deposited to NCBI database (GenBank accession number, OR039202) was totally 161,352 bp in length, containing four typical regions as a large single-copy region (LSC, 89,802 bp), a small single-copy region (SSC, 18,914 bp), and two identical inverted repeat regions (IR, 26,318 bp). The overall GC content of cp genome of *S. thea* was 37.10%. A total of 129 functional genes were identified, including 8 rRNA genes, 37 tRNA genes and 84 protein-coding genes (Figure 2). There were 15 genes that display one intron (including *atpF, ndhA, ndhB, petB, petD, rpl16, rpl2, rps16, rpoC1, trnA-UGC, trnG-UCC, trnI-GAU, trnK-UUU, trnL-UAA,* and *trnV-UAC*); three genes (*clpP, ycf3,* and *rps12*) contained two introns in the cp genome of *S. thea*. One *trans*- and 13 *cis*-splicing genes were annotated manually, including *rps12, rps16, atpF, rpoC1, ycf3, clpP, petB, petD, rpl16, rpl2* (two copies), *ndhB* (two copies), and *ndhA* (Figure S2A,B). The average read coverage depth map for the cp genome of *S. thea* assembly reached 2753× (Figure S2C). The phylogenetic results showed that the cp genome of *S. thea* is closely related to four-genus of Rhamnaceae family, including *Rhamnus, Berchemiella, Rhamnella* and *Berchemia* (Figure 3).

## Discussion and conclusion

Due to their high-throughput, time-saving, and cost-effectiveness, Next- and Third-generation sequencing technologies have gradually gained popularity in genomic research (Cronn et al. 2008). Although the Rhamnaceae family comprises approximately 900 species, there is a limited availability of genomic sequences for this taxonomic group (Ma et al. 2017). Phylogenetic analysis of representative species from different genera within Rhamnaceae revealed a close relationship between the cp genome of *S. thea* and those of *Rhamnus*, *Berchemiella*, *Rhamnella*, and *Berchemia*; however, it showed distant relatedness to *Ziziphus. Ziziphus*, as a member of the Rhamnaceae family, is widely distributed in subtropical and tropical regions of Asia and America. On the other hand, *S. thea* exhibits wide distribution in subtropical regions of Asia. Therefore, we hypothesize that geographical isolation exists between *S. thea* and *Ziziphus* species leading to significant genetic diversity differences (Ma et al. 2017).

The chloroplast genome of *S. thea* was detected and analyzed in this study, providing essential insights for the conservation, utilization, and phylogenomic investigations of the Rhamnaceae family.

## Supplementary Material

Supplemental Material

Supplemental Material

Supplemental Material

## Data Availability

The genome sequence data that support the findings of this study are openly available in GenBank of NCBI at [https://www.ncbi.nlm.nih.gov] under the accession number of OR039202. The associated BioProject, SRA, and Bio-Sample numbers are PRJNA983071, SRR24916185, and SAMN35719156.
