# Supplementary material for: Complete chloroplast genome of sageretia thea (rhamnaceae), an ornamental fruit and medicinal tree
Source: Mitochondrial DNA B Resour. 2024 Mar 26;9(3):376–80. doi: 10.1080/23802359.2024.2329667 (PMC10967667; doi:10.1080/23802359.2024.2329667)
Supplement: Supplemental Material [file TMDN_A_2329667_SM1998.docx]

Supplement Figure S1 The dotplot collinearity with reference sequence of the assembly of the cp genome of *S. thea*. The X-axis is the reference sequence, the Y-axis is the assembly sequence, the black line represents the unique alignment information, the red line represents the multi-alignment information, the positive triangle represents cis-collinearity, and the inverse triangle represents inverse collinearity; This figure shows the conserved and rearranged segments of the genome, the longer the diagonal, the less rearrangement, and the more conserved the segments.

Supplement Figure S2 Cis-, trans-splicing genes and read coverage depth map of the assembly of the cp genome of *S. thea*. .(A, The structure of the 13 protein-coding cis-splicing genes annotated in cp genome of *S. thea*; B, The structure of the 1 protein-coding trans-splicing genes annotated in cp genome of *S. thea*; C, The read coverage depth map of the assembly of the cp genome of *S. thea*; The numbers in the picture A and B represent the location in the Cp genome of *S. thea*; The figure C means the coverage depth on each base of chloroplast genome of *S. thea,* X-axis indicates the location of bases in the genome; Y-axis indicates the coverage depth.)
