# Supplementary figures and images for: Complete chloroplast genome of sageretia thea (rhamnaceae), an ornamental fruit and medicinal tree
Source: Mitochondrial DNA B Resour. 2024 Mar 26;9(3):376–80. doi: 10.1080/23802359.2024.2329667 (PMC10967667; doi:10.1080/23802359.2024.2329667)

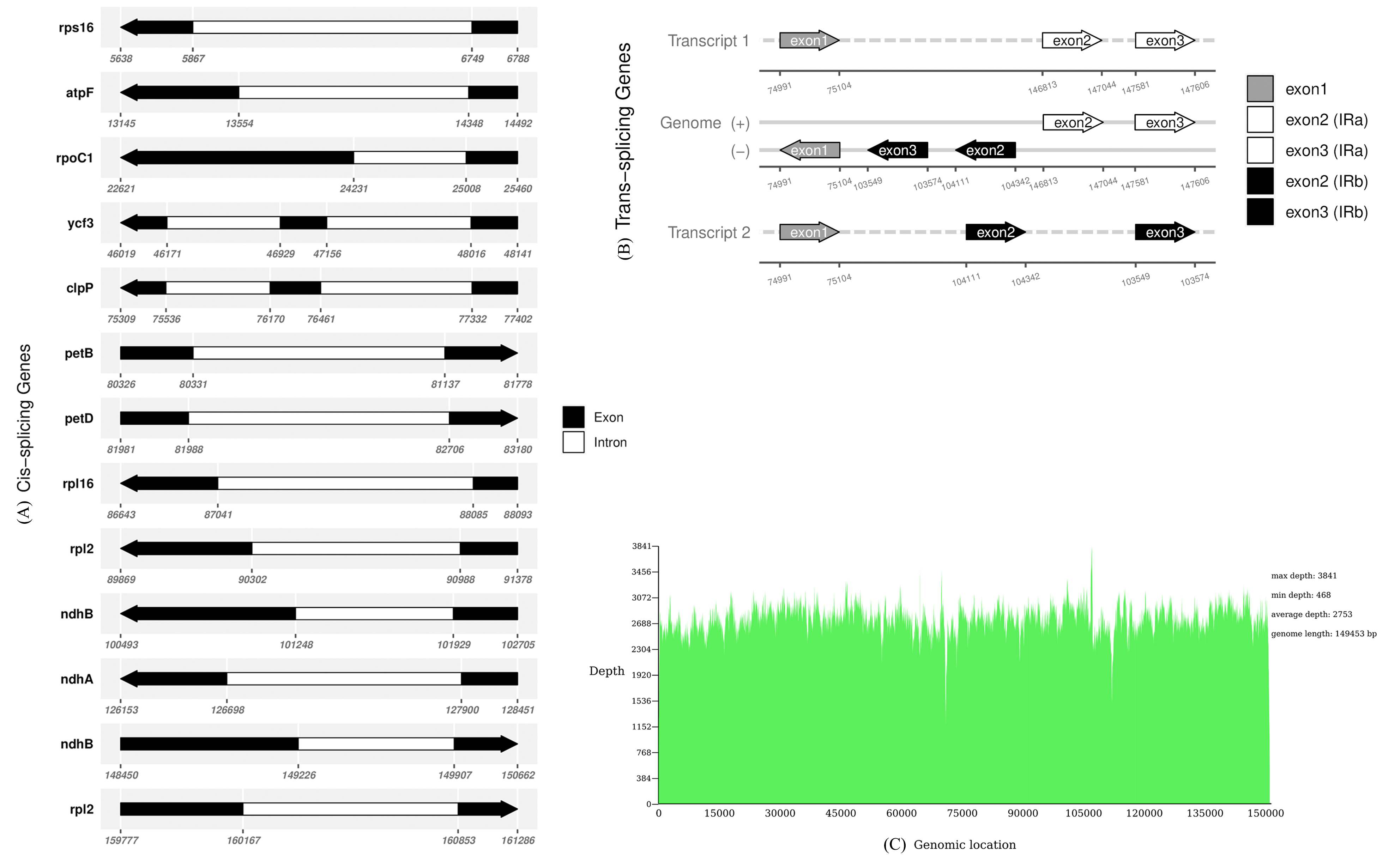

Supplement: Supplemental Material [file TMDN_A_2329667_SM1991.jpg]

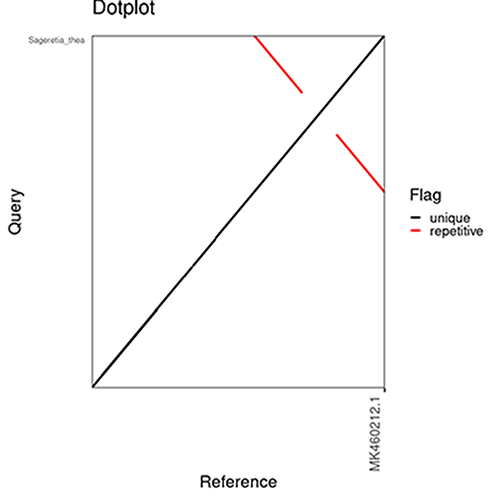

Supplement: Supplemental Material [file TMDN_A_2329667_SM1987.jpg]
